# Transcriptome difference and potential crosstalk between liver and mammary tissue in mid-lactation primiparous dairy cows

**DOI:** 10.1371/journal.pone.0173082

**Published:** 2017-03-14

**Authors:** Dengpan Bu, Massimo Bionaz, Mengzhi Wang, Xuemei Nan, Lu Ma, Jiaqi Wang

**Affiliations:** 1 State Key Laboratory of Animal Nutrition, Institute of Animal Science, Chinese Academy of Agricultural Sciences, Beijing, P.R. China; 2 CAAS-ICRAF Joint Laboratory on Agroforestry and Sustainable Animal Husbandry, World Agroforestry Centre, East and Central Asia, Beijing, China; 3 Synergetic Innovation Center of Food Safety and Nutrition, Harbin, China; 4 Department of Animal and Rangeland Sciences, Oregon State University, Corvallis, United States of America; 5 College of Animal Science and Technology, Yangzhou University, Yangzhou, Jiangsu Province, P.R. China; Wageningen UR Livestock Research, NETHERLANDS

## Abstract

Liver and mammary gland are among the most important organs during lactation in dairy cows. With the purpose of understanding both the different and the complementary roles and the crosstalk of those two organs during lactation, a transcriptome analysis was performed on liver and mammary tissues of 10 primiparous dairy cows in mid-lactation. The analysis was performed using a 4×44K Bovine Agilent microarray chip. The transcriptome difference between the two tissues was analyzed using SAS JMP Genomics using ANOVA with a false discovery rate correction (FDR). The analysis uncovered >9,000 genes differentially expressed (DEG) between the two tissues with a FDR<0.001. The functional analysis of the DEG uncovered a larger metabolic (especially related to lipid) and inflammatory response capacity in liver compared with mammary tissue while the mammary tissue had a larger protein synthesis and secretion, proliferation/differentiation, signaling, and innate immune system capacity compared with the liver. A plethora of endogenous compounds, cytokines, and transcription factors were estimated to control the DEG between the two tissues. Compared with mammary tissue, the liver transcriptome appeared to be under control of a large array of ligand-dependent nuclear receptors and, among endogenous chemical, fatty acids and bacteria-derived compounds. Compared with liver, the transcriptome of the mammary tissue was potentially under control of a large number of growth factors and miRNA. The *in silico* crosstalk analysis between the two tissues revealed an overall large communication with a reciprocal control of lipid metabolism, innate immune system adaptation, and proliferation/differentiation. In summary the transcriptome analysis confirmed prior known differences between liver and mammary tissue, especially considering the indication of a larger metabolic activity in liver compared with the mammary tissue and the larger protein synthesis, communication, and proliferative capacity in mammary tissue compared with the liver. Relatively novel is the indication by the data that the transcriptome of the liver is highly regulated by dietary and bacteria-related compounds while the mammary transcriptome is more under control of hormones, growth factors, and miRNA. A large crosstalk between the two tissues with a reciprocal control of metabolism and innate immune-adaptation was indicated by the network analysis that allowed uncovering previously unknown crosstalk between liver and mammary tissue for several signaling molecules.

## Introduction

Mammary gland is an important organ in mammals. When lactation starts the mammary gland becomes an extremely anabolic organ in order to produce and secrete large amount of milk rich in lactose, lipids, and proteins. The mammary gland of a normal high producing dairy cow can produce up to 2 kg/d of lactose, 1.3 kg/d of protein, and 1.6 kg/d of fat. It has been estimated that a dairy cow at peak lactation can produce almost 1 kg/d of milk per each g of mammary epithelial DNA [1]. The extremely large anabolic capacity of the mammary gland of dairy cows during lactation requires a large amount of nutrients, particularly energy and proteins. The increase in energy and protein requirement of the mammary gland in dairy cows is approx. 5-fold from late gestation to lactation [2]. The large anabolic change in the mammary tissue from pregnancy to lactation is mainly due to increase in cellular activity which is evidenced by a 3-fold increase in synthesis of RNA [3] and a 4-fold increase in mRNA translation [4].

The organ that plays a central role in supporting the anabolic capacity of the mammary gland is the liver. In ruminants, >90% of the glucose available for all functions is produced *de novo*. Many tissues can perform gluconeogenesis; however, the liver contributes for >80% of the glucose produced via gluconeogenesis, as demonstrated in sheep [5], [6]. Besides gluconeogenesis, the liver plays a central role in lipid metabolism, amino acid metabolism, detoxification, and immune defense [7], [8].

The liver and the mammary tissue differ substantially considering the dissimilar embryonic origin (endodermic for the liver and ectodermic for the mammary), function (mammary produces milk while the liver is an essential organ for metabolism, clearance, and inflammatory response, among others), and metabolic status where the liver is more catabolic and mammary tissue is more anabolic.

Any physiological adaptation required a concerted activity of several organs/tissues. The physiological adaptation to lactation requires coordinated change in activity of the tissues composing the whole organism. The coordinated adaptation, called homeorhesis, is mostly driven by hormones and tissue sensitivity and is a relatively gradual adaptation [9]. The hormonal regulation of lactation is relatively well-known and appears to be mostly driven by the hypothalamus-hypophysis axis [10]; however, the direct crosstalk between the various organs remains poorly known. Knowledge on the crosstalk of biological pathways at gene expression level between tissues and organs, especially the crosstalk between liver and mammary, is very important to fully understand the factors influencing milk production. Inter-tissue crosstalk is an important factor in controlling health and disease in monogastrics [11] and development, as observed between parenchyma and fat pad in the development of the mammary tissue in calves [12].

Crosstalk between tissues is determined by the interactions between secreted molecules from one tissue with the related receptor(s) in the other tissue(s). The transcriptome data can allow identifying indirectly the relative abundance of both signaling proteins and receptors in a tissue-specific manner. The use of transcriptomics data to study crosstalk between tissues has been used previously with success [11], [12], [13].

The purpose of this study was to understand both the difference and the complementary roles of liver and mammary tissue during lactation, the upstream factors participating in controlling the transcriptome difference, and the potential crosstalk between the two tissues by functional analysis of genes differentially expressed between the two tissues in mid-lactation primiparous dairy cows.

## Material and methods

### Ethics statement

This study was specifically approved by the Animal Care and Use Committee of the Institute of Animal Science, Chinese Academy of Agricultural Sciences. The use of animals in the present study was in strict accordance with the Directions for Caring of Experimental Animals from the Institute of Animal Science, Chinese Academy of Agricultural Sciences.

### Samples collection and preparation

Liver and mammary tissue samples were obtained by biopsy from 10 primiparous lactating Holstein cows (body weight, 558 ± 10 kg; days in milk, 136 ± 37d; daily milk yield, 21.1 ± 2.3 kg) as previously described [3], [14]. These cows were a subset (5 cows from each treatment group) of a larger study [15]. The cows used in the present experiment had no mastitis, were pregnant and in their first lactation.

Throughout the experiment, cows were housed in a tie stall barn, and diet was formulated to meet the requirements according to the National Research Council (2001) (S1 Table).The diet was mixed daily and fed *ad libitum* as total mixed ration. The cows were fed twice per day at 07:00 and 19:00 h in an equal amount that allowed for 10% residuals. Cows were milked twice daily at 07:00 and 19:00 h and had free access to water. The liver and mammary biopsies were performed simultaneously (i.e., within 40 minutes) at approximately 0700 h (post-AM milking). The cows received a small dose of xylazine (0.05 mg/kg BW) before applying a local anesthesia. Prior to the incision, 3 to 4 mL of lidocaine-hydrochloride (2% solution) was injected subcutaneously as local anesthetic. For the mammary biopsy, a 3-cm incision using a sterile scalpel blade was performed on the midsection of left rear quarter. The parenchyma tissue was removed and the mammary epithelium exposed. Once the parenchyma was visible a biopsy was performed using a cordless drill equipped with a bioptic probe (AgResearch Ruakura, Ruakura Agricultural Center, Hamilton, New Zealand, 85 mm in length by 4.5 mm in diameter). Immediately after removal of the biopsy instrument, we applied pressure to stop bleeding using sterilized gauze. Approx. 400 mg of mammary tissue was obtained from the biopsy. The liver tissue (around 300 mg) was collected via puncture biopsy. A parenchyma area far from the large hepatic blood vessels was identified using a 3.5 MHz ultrasound probe, and chosen as the site for biopsy. A 1.5 cm incision using a sterile scalpel blade was done between the 11^th^ and 12^th^ rib on the right side of cow. Following the skin incision proper pressure with sterile gauze was applied to the wound until visual signs of bleeding were absent. The biopsy of the liver was performed using a Tru-Cut biopsy needle (Tru-Cut Biopsy Needle, Baxter Healthcare Corp., Valencia, CA, USA, diameter 4 mm). For both biopsies, the skin incision was closed with 4 or 5 Michel clips (11 mm; Henry Schein, Melville, NY, USA). The incision site was sprayed with topical antiseptic (10% Povidone Iodine Ointment; Taro Pharmaceuticals, Hawthorne, NY, USA). Health was monitored post-surgery by recording rectal temperature, milk yield, and feed intake daily for 7 days. Surgical clips were removed 7 days post-biopsy. These cows were a subset of a larger study [15], hence, after biopsy they were placed back to their tie stall barns until completion of that study.

Tissue samples were washed with PBS buffer prepared with RNase, DNase-free water, hydrated, and immediately stored in liquid nitrogen until RNA extraction.

### RNA extraction and microarray

Total RNA was extracted with TRIzol reagent (Life technologies, US, Cat#74106) according to the manufacturer’s protocol. The total RNA was purified by Rneasy mini kit (QIAGEN, Germany, Cat#74106) and RNase-Free DNase Set (QIAGEN, Germany, Cat#79254). The concentration was measured by NanoDrop1000. The OD260/OD280 values were ≥ 1.9. Integrity of the purified total RNA was assessed using 2100 Bioanalyzer (Agilent Technologies, US) and the RNA 6000 Nano Kit (Agilent Technologies, US). The RIN (RNA Integrity Number) values were ≥ 8.0.

Transcriptomic analysis was performed using 4×44K Bovine microarray chip (Agilent Technologies, US, design ID: 023647) with the capacity to measure 17,252 unique annotated genes. Total RNA was amplified and labeled by Low Input Quick Amp Labeling Kit, One-Color (Agilent technologies, US Cat#5190–2305) according to the manufacturer’s instructions. Each slide was hybridized with 1.65 μg Cy3-labeled cRNA using Gene Expression Hybridization Kit (Agilent technologies, US, Cat#5188–5242,) in an hybridization oven (Agilent technologies, US). After 17 hours hybridization slides were washed in staining dishes (Thermo Shandon, US) with Gene Expression Wash Buffer Kit (Agilent technologies, US, Cat#5188–5327).

Slides were scanned by Agilent Microarray Scanner (Agilent technologies, US) with default settings (i.e., dye channel: Green, Scan resolution = 5 μm, PMT, 100%, 10%, 16bit). Data was acquired with Feature Extraction software 10.7 (Agilent technologies, US). The microarray dataset presented in this manuscript was deposited at NCBI's Gene Expression Omnibus and is accessible through GEO Series accession number GSE73980.

### Quantitative real-time RT-PCR (RTqPCR)

The RNA was reverse-transcribed to cDNA using the High-capacity cDNA Reverse Transcription Kit (4368814, Applied Biosystems, Carlsbad, America). cDNA was amplified with Power SYBR^®^ Green PCR Master Mix (4367659, Applied Biosystems, Carlsbad, America) using the Applied Biosystems (ABI) 7500 PCR machine. Final RTqPCR data were obtained by 2^-ΔΔCt^ method. Six potential internal control genes were tested (*GAPDH*, *PEX5*, *POLR2H*, *S100A10*, *SELL*, and *MTG1*) using geNorm [16]. The best normalization factor was obtained by using 5 ICG (all above genes except *MTG1*; V-value = 0.197) showing in S5 File. Eight genes (*ABCG2*, *ACOX1*, *APOB*, *C3*, *CSN2*, *FABP3*, *FABP4*, and *LPL*) were analyzed to compare with the microarray data. Primer-pairs sequences are reported in S6 File.

### Statistical analysis

Raw data were normalized by quantile method with Gene Spring Software 11.0 (Agilent technologies, US) and uploaded into JMP Genomics (SAS institute, NC, USA) for statistical analysis. Data were log_2_ transformed and values of the annotated genes with multiple oligos were averaged before statistical analysis. Biological outliers were analyzed using JMP Genomics. None of the animals were considered outliers, as demonstrated by the Outlier Box Plots (S1 Fig). Tissue and Treatment×Tissue effect were assessed using ANOVA and using animal as random effect. The effect of treatment was not part of the present analysis but the Treatment×Tissue effect was performed to assess if any gene affected by treatment had an effect on the difference detected between tissues. The effect of treatment was nil (FDR<0.001 and P-value<0.001) on genes detected to be differentially expressed between tissues. A false discovery rate correction [17] was applied.

#### Functional analysis of microarray data

The analysis of the Kyoto Encyclopedia of Genes and Genomes (KEGG) pathways was performed by the Dynamic Impact Approach (DIA) [18]. For the DIA analysis all Entrez Gene ID of the microarray were used as background and the whole dataset with Entrez Gene ID, FDR, expression ratio, and P-value was uploaded. A FDR<0.001 and a P-value <0.001 between the two tissues were used as cut-off.

The enrichment analysis of various database including KEGG pathways, Gene Ontology Biological process and Cellular components was run by Database for Annotation, Visualization and Integrated Discovery (DAVID) v6.7 [19] in combination with REVIGO [20]. For the purpose the whole annotated microarray (Entrez Gene ID) was used as background and four datasets were analyzed: 1) dataset encompassing all DEG more expressed in liver *vs*. mammary; 2) dataset encompassing all DEG ≥2-fold more expressed in liver *vs*. mammary; 3) dataset encompassing all DEG more expressed in mammary *vs*. liver; 4) dataset encompassing all DEG ≥2-fold more expressed in mammary *vs*. liver. The default database present in DAVID plus the UP-tissue were used (the analysis was run in June 2014). Results were downloaded using the Functional Annotation Chart.

The enrichment analysis of pathways, functions, and upstream regulators were performed using Ingenuity Pathway Analysis (IPA; Ingenuity Systems, CA, USA). The whole dataset containing Entrez Gene ID, FDR, expression ratio, and P-value for the comparison between liver and mammary tissue was uploaded into IPA and the whole annotated microarray was used as background. Due to the nature of the sample analyzed the IPA analysis was restricted to IPA database related to liver as organ system and all Hepatoma cell lines for the data related to liver and IPA dataset related to mammary gland as organ system and Breast Cancer cell lines and Other cell lines for the data related to mammary tissue. A cut-off of FDR≤0.001, fold change of 1 and P-value≤0.001 were set for the analysis.

#### Upstream regulator analysis

Ingenuity Pathway Analysis was used to analyze the upstream regulators of DEG using the “Upstream analysis” feature as previously described [13]. The analysis uses an IPA Knowledge base in combination with the data of the target genes (i.e., the DEG in our analysis) to predict up-stream regulators. Besides identifying the up-stream regulators, the analysis provides the more plausible prediction of the status of the upstream regulators (i.e., activated or inhibited) or the effect on the down-stream genes (i.e., activating or inhibiting) by computing an activation Z-score. The results were downloaded from IPA and graphical depicted using SigmaPlot v11 (Systat Software Inc., Germany).

#### Crosstalk analysis

The crosstalk between liver and mammary tissue was performed using the network capability of IPA as previously described [12][13]. For the present analysis DEG considered to code for secreted proteins were the one in the cytokines and growth factors categories while genes coding for proteins considered to be receptors (thus, able to “sense” the secreted proteins) were the ones in G-protein coupled receptor, ligand-dependent nuclear receptor, transcription regulator, and transmembrane receptor categories. Networks between DEG with high expression in liver *vs*. mammary and coding for secreted proteins and DEG more expressed in mammary tissue and coding for receptors and *vice versa* were built using IPA Knowledge base.

## Results and discussion

### Differentially expressed genes (DEG) between liver and mammary tissue

**Fig 1:**
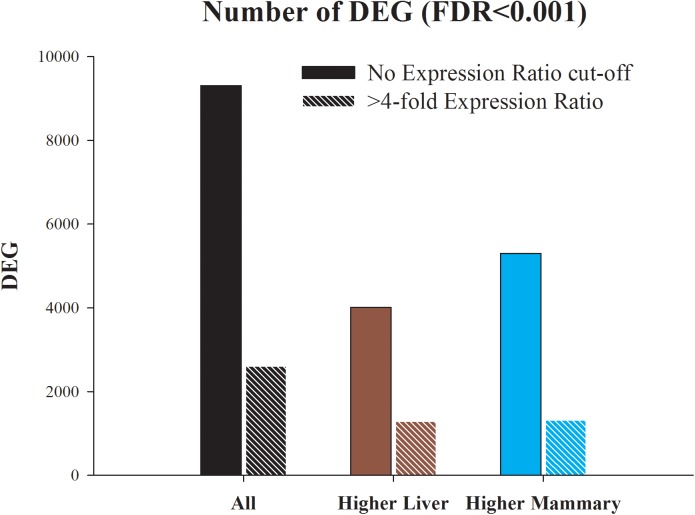
Number of differentially expressed genes (DEG; Benjamini & Hochberg false discovery rate or FDR≤0.001) between liver and mammary tissue obtained from mid-lactation primiparous dairy cows. Shown are all DEG, the DEG more expressed in liver compared with mammary tissue, and the DEG more expressed in mammary tissue compared with liver. Presented are also the DEG with >4-fold expression ratio between the two tissues.

The number of DEG is summarized in Fig 1 and an overview of the scatter plot of transcriptomic difference between mammary and liver tissue is available in S2 Fig while in S3 Fig are reported the top 60 DEG. In S1 File is reported the whole dataset with statistical results. The results from the current work only represent a 40 minute snapshot of the liver and mammary gland transcriptome following feeding and milking.

The use of a FDR<0.001 resulted in >9,000 DEG between liver and mammary tissue (>55% of all annotated genes, Fig 1). When a 4-fold expression ratio cut-off was applied there were still >2,600 DEG between the two tissues (>15% of annotated genes). There was a higher number of DEG more expressed in mammary compared with the one more expressed in liver but the DEG more expressed in liver compared to mammary had a higher average of expression ratio (154 vs. 19 or geometrical mean 3.6 vs. 1.6; S1 File). Eight genes detected to be differentially expressed with the microarray analysis were selected and validated by RTqPCR (S4 Fig).

Among the DEG with the highest expression ratio in liver compared to mammary there were some classical liver-specific genes, such as *FABP1*, *APOH*, *ALB*, and *PON1* but also some less known genes, such as vitamin D binding protein (*GC*) and vitronectin (*VTN*) (S3 Fig). Approximately half of the 30 DEG with the highest expression ratio in liver *vs*. mammary tissue are secreted by the liver into the blood stream and several are involved in the acute phase reaction (e.g., *ALB*, *OSM1*, and two *SERPINA* isoforms) (S3 Fig).

Among the 30 DEG more expressed in mammary tissue compared to liver we observed some of the classical mammary-specific genes, such as the 4 caseins, *LALBA*, *FABP3*, and *BTN1A1* but the DEG with the highest expression in mammary compared to liver were glycosylation-dependent cell adhesion molecule-1 (*GLYCAM1*), a protein involved in lymphocytes extravasation but with likely other functions in mammary tissue [21], fibroblast growth factor-binding protein 1 (*FGFBP1*), which plays a critical role in cell proliferation, differentiation and migration by binding to fibroblast growth factors receptor [22], the cysteine-rich secretory protein 3 (*CRISP3*), and G protein-coupled receptor 68 (*GPR68*). For the last two genes a function of the coded proteins in mammary tissue has not been discovered yet.

Overall the data indicate a very large transcriptomic difference between the liver and mammary tissue in mid-lactation dairy cows. This difference is not so surprising considering the different embryonic origin (endodermic for liver and ectodermic for mammary) and completely different biological tasks between mammary tissue and liver.

### Overall view of KEGG pathways difference between liver and mammary tissue using DIA

**Fig 2:**
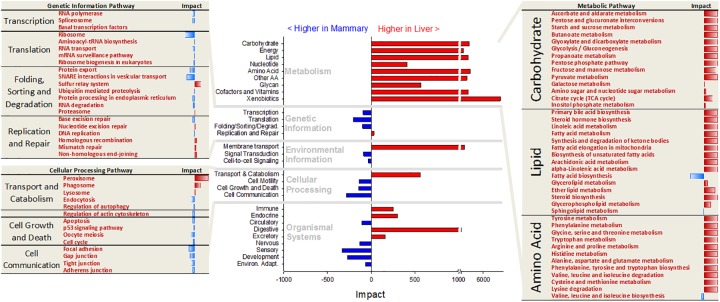
Direction of the impact of main sub-categories of KEGG pathways (in the center, with main categories of pathways reported in light grey font) and most impacted pathways in the ‘Genetic Information processing’, ‘Cellular Processing’, and ‘Metabolism’ subcategories of pathways. For the ‘Metabolism’ subcategories of pathways only the one related to carbohydrate, lipid, and amino acid metabolism are shown (red font = more activated in liver and blue font = more activated in mammary tissue).

The Dynamic Impact Approach (DIA) is a novel method which can provide an estimation of the biological impact and the direction of the impact for a given set of genes [18]. We conducted DIA analysis to determine the differences in KEGG pathways between the two tissues (Fig 2 and more details in S2 File).

#### Metabolic pathways

The results uncovered a larger induction in liver *vs*. mammary tissue of almost all metabolic pathways (Fig 2). All the pathways in the ‘Carbohydrate metabolism’ category of pathways were more induced in liver compared to mammary tissue. Surprisingly, also the ‘Galactose metabolism’ pathway was slightly more induced in the liver compared with mammary tissue. However, a detailed visualization of the pathway (S5 Fig) revealed, as expected, a larger induction of lactose synthesis in mammary tissue and the overall slightly larger induction in liver vs. mammary tissue of this pathway was due to the larger expression in liver vs. mammary of genes involved in galactose degradation. All the ‘Amino acid metabolism’ pathways with exception of ‘Valine, Leucine, and Isoleucine biosynthesis’ were more induced in liver compared to mammary tissue. The liver is known to be the main site of nitrogen metabolism in ruminants [23] and part of the amino acids in the liver are used for the synthesis of glucose [6].

The xenobiotic metabolism was the most different between the two tissues and with a larger induction in liver compared with mammary tissue (Fig 2). The liver is known to be the main site of xenobiotics metabolism and elimination. The mammary gland has also a large capacity for transport and elimination of xenobiotics through the milk [24]. The mammary tissue presents a metabolism of xenobiotics but at substantially lower level compared with the liver [25].

The results from the DIA analysis of the lipid metabolism-related pathways is indicative of a liver that, not only oxidizes more fatty acids which is considered to be the predominant lipid metabolism in liver of lactating cows [26], but, surprisingly, compared with mammary tissue the liver had also a higher induction of pathways involved in the synthesis of triacylglycerol (e.g., ‘Biosynthesis of unsaturated fatty acids’ and ‘Glycerolipid metabolism’), phospholipids (e.g., ‘Glycerophospholipid metabolism’), cholesterol (i.e., ‘Steroid biosynthesis’), and leukotrienes (through ‘Arachidonic acid metabolism’) (Fig 2 and S2 File). Those data are somewhat contrasting with previous data in mouse [27] and ruminants [28] where a higher lipogenic capacity was observed for mammary tissue compared with liver. This is even more striking considering that in ruminants the lipogenic capability of the liver is lower than rodents [29]. However, among lipid metabolism, the *de novo* fatty acid synthesis was more induced in mammary tissue compared with the liver (Fig 2). This is consistent with the large capability of mammary tissue for the production of approx. half of the milk fat starting from acetate and butyrate [30].

The DIA analysis of the non-metabolic-related pathways revealed a larger transcription and protein synthesis capability in mammary tissue *vs*. liver among pathways related to ‘Genetic Information processing’ (Fig 2). The liver had a larger induction of pathways related to membrane transporters (i.e., “ABC transporters”, see S2 File) but mammary tissue had a larger induction compared with liver of signaling-related pathways (Fig 2).

Among cellular processing-related pathways, the liver had an overall larger induction compared with mammary tissue of transport and catabolism, mostly due to peroxisomes, while mammary tissue had a larger induction compared with liver of cell cycle, cell motility, and cell communication (i.e., focal, gap, and tight junctions) (Fig 2 and S2 File). Considering organismal system-related pathways the liver had an overall higher induction of pathways related to immune response almost exclusively due to a very large impact and induction in liver *vs*. mammary tissue of complement and coagulation cascades. A more detailed view of the pathways, however, clearly uncovered that most of the immune-related pathways were instead more induced in mammary tissue *vs*. liver (see S2 File). In particular, receptor signaling pathways (e.g., Toll-like, RIG-I-like, NOD-like, and immune cells receptors) and “Leukocyte transendothelial migration” were more induced in mammary tissue compared to liver. Data indicate that the liver is more prone to an acute phase response, which has been clearly demonstrated by many works done in several mammals and confirmed in dairy cows (e.g.,[31]), but the mammary is more involved in preparing a coordinated immune response. The latter was also suggested by other transcriptomic data from bovine mammary tissue [32].

Among endocrine related pathways the ‘PPAR signaling’ was the most impacted and more induced in liver *vs*. mammary tissue while “Renin-angiotensin system” was more induced in mammary tissue *vs*. liver (S2 File). The insulin and adipocytokine signaling pathways were more induced in liver *vs*. mammary tissue (S2 File). A detailed visualization of the above pathways (S6–S8 Figs) delineated a picture where mammary tissue *vs*. liver is more sensitive to insulin signaling (S6 Fig). Particularly, the data indicated that, in mammary tissue compared with liver, insulin had a higher control of lipogenesis, protein synthesis, and proliferation while in liver insulin had a higher control of glycogenesis [33], [34]. An important role of insulin in the control of milk synthesis, particularly milk protein synthesis, has emerged in the last decade or so [35]. The pivotal role of insulin in the control of liver glycogenesis, a prominent function of the liver, has been established several decades ago [36]. Despite an overall more induced ‘PPAR signaling’ in liver *vs*. mammary tissue, the detailed analysis of the data clearly indicated that compared with liver the mammary tissue had an overall higher PPAR sensitivity (due to higher expression of retinoic X receptor [RXR], the protein essential for the formation of active PPAR heterodimer; see S1 File) but also higher expression and activity of PPARγ and PPARβ/σ, more related to lipogenesis, while in liver *vs*. mammary tissue the PPARα was more prominent with a control of fatty acid oxidation and bile, glycerophospholipids, gluconeogenesis, and ketones metabolism (S7 Fig). These data confirmed the well-established lipid and glucose metabolism role of the liver [26] and seem to support recent data on the role of the other two PPAR isotypes in mammary tissue of dairy cows [37]. The detailed visualization of the adipocytokines signaling pathway (S8 Fig) revealed higher insulin signaling control of mammary tissue *vs*. liver but also a higher sensitivity of the mammary toward adiponectin, indicating a more important crosstalk between adipose and mammary tissue for the control of glucose uptake, while the liver appeared to be more sensitive to leptin (i.e., higher leptin receptor expression; S8 Fig). The above observations confirm previous data [8], [38].

The mammary tissue had a higher induction compared with liver of pathways related to circulatory, sensory, and development (Fig 2). The suggested larger blood circulation in lactating mammary tissue *vs*. liver is in accordance with the extremely large increase in vessels and blood circulation from non-lactating to lactating mammary tissue [39].

### Gene Ontology analysis confirmed DIA results

The KEGG database present established metabolic and signaling pathways, but only a relatively low number of genes are included in the KEGG pathway database. Gene Ontology (GO), a larger and more integrated database, can provide a more holistic view of the results (S3 File).

The GO Biological process analysis results further confirmed the stronger metabolic capacity of liver *vs*. mammary tissue as suggested by the DIA analysis of KEGG pathways, particularly for lipids, immune response (i.e., acute phase reaction, complement activation), and detoxification (S9 Fig). The mammary tissue *vs*. liver had a larger developmental capacity, signal transduction, endocytosis, and ECM organization (S9 Fig).

Results from the GO Cellular components analysis revealed a higher enrichment of genes coding for mitochondria components and several microsomes (e.g., peroxisome) in DEG more expressed in liver *vs*. mammary tissue but also highly enriched were genes related to secreted proteins (S10 Fig), while DEG more expressed in mammary tissue *vs*. liver highly enriched terms related to plasma membrane (particularly basolateral membrane), ribosome, ECM, and components of the cell junctions.

Overall the GO results confirmed the DIA analysis of KEGG pathways and clearly differentiated the two tissues, with the liver having a higher metabolic capacity compared with the lactating mammary tissue and the mammary tissue having a larger communication capacity and trafficking of membranes.

### Most enriched pathways and functions in Ingenuity Pathway Analysis tool

**Fig 3:**
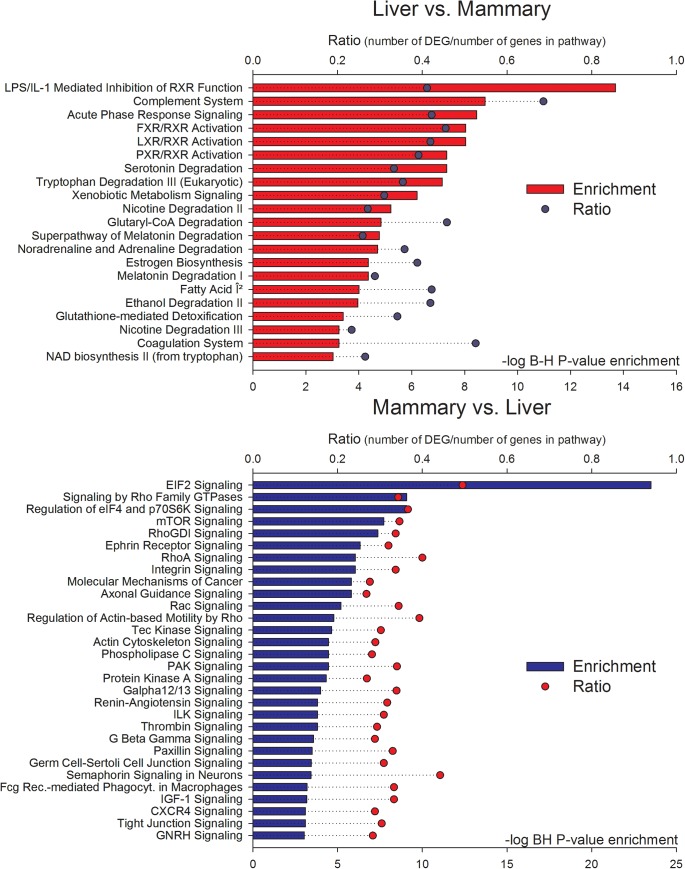
Significant (Benjamini & Hochberg FDR [B-H]<0.001 or–log_10_ B-H>3) enriched pathways in DEG more expressed in liver *vs*. mammary tissue (top panel with red horizontal bars) and in DEG more expressed in mammary tissue *vs*. liver (bottom panel in blue horizontal bars). The round symbols denote the ratio of DEG compared with all genes in the pathway. Results are from Ingenuity Pathway Analysis.

Most enriched pathways in DEG between liver and mammary tissue uncovered by Ingenuity Pathway Analysis (IPA) are reported in Fig 3 and S4 File. The IPA analysis uncovered a significant enrichment of metabolic-related and acute phase-related pathways among DEG more expressed in liver *vs*. mammary tissue. In DEG more expressed in mammary tissue *vs*. liver the most enriched pathways were all signaling-related. A more in-depth analysis revealed in DEG more expressed in liver *vs*. mammary tissue a large enrichment of ligand-dependent nuclear receptor-related pathways, particularly involving the nuclear receptors Liver X Receptor (LXR), Farsenoid X receptor (FXR), and Pregnane X receptor (PXR). In addition, the analysis indicated a larger importance in liver *vs*. mammary tissue of metabolic pathways involved in degradation of components, such as amino acids, fatty acids, and melatonin, and a strong link between inflammatory response and regulation of metabolism through the RXR (i.e., “LPS/IL1 mediated inhibition of RXR function”).

The analysis revealed a larger importance of protein synthesis regulation in mammary tissue compared with liver (i.e., EIF2 and mTOR signaling pathways; Fig 3) but also a larger importance of cell-to-cell communication and cytoskeleton organization (i.e., integrin, tight junctions, and actin cytoskeleton signaling pathways).

**Fig 4:**
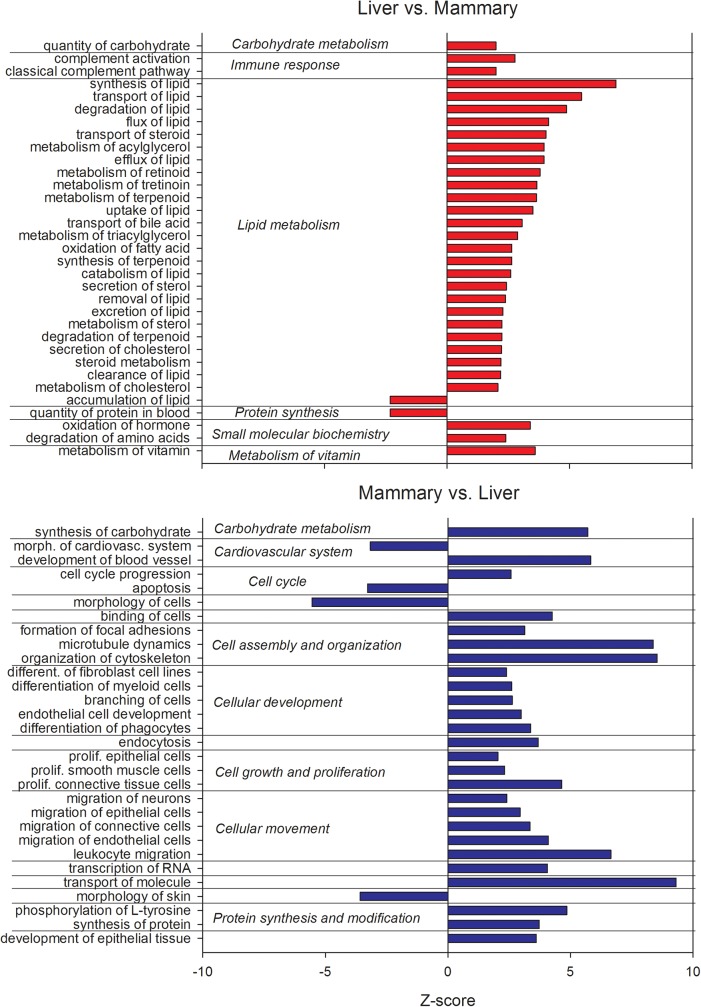
Function with a Z-score ≥2 of DEG more expressed in liver *vs*. mammary tissue (upper panel) and DEG more expressed in mammary tissue *vs*. liver (lower panel). The Z-score is an estimate of the activation or inhibition of the function based on the expression of the DEG related with the function and the known effects of the DEG on the function. Results were obtained using Ingenuity Pathway Analysis.

Among functions (Fig 4 and S4 File) highly enriched by DEG and more expressed in liver *vs*. mammary tissue were genes related to lipid metabolism (all more induced in liver with exception of “accumulation of lipids”), including vitamin metabolism, while protein synthesis appeared to be more induced in mammary tissue *vs*. liver. Among DEG more expressed in mammary tissue *vs*. liver there was a significant enrichment and activation of functions related to proliferation, development, cell assembly and organization, cell movement, and RNA and protein synthesis, while apoptosis was enriched but inhibited (Fig 4).

All the above clearly support the findings from the DIA and DAVID analyses and provides an additional case for a stronger metabolic and inflammatory signature in liver compared to mammary tissue and a stronger protein synthesis, development, proliferation, and communication signature in mammary tissue compared to liver.

### Upstream regulators defining the difference between liver and mammary tissue

**Fig 5:**
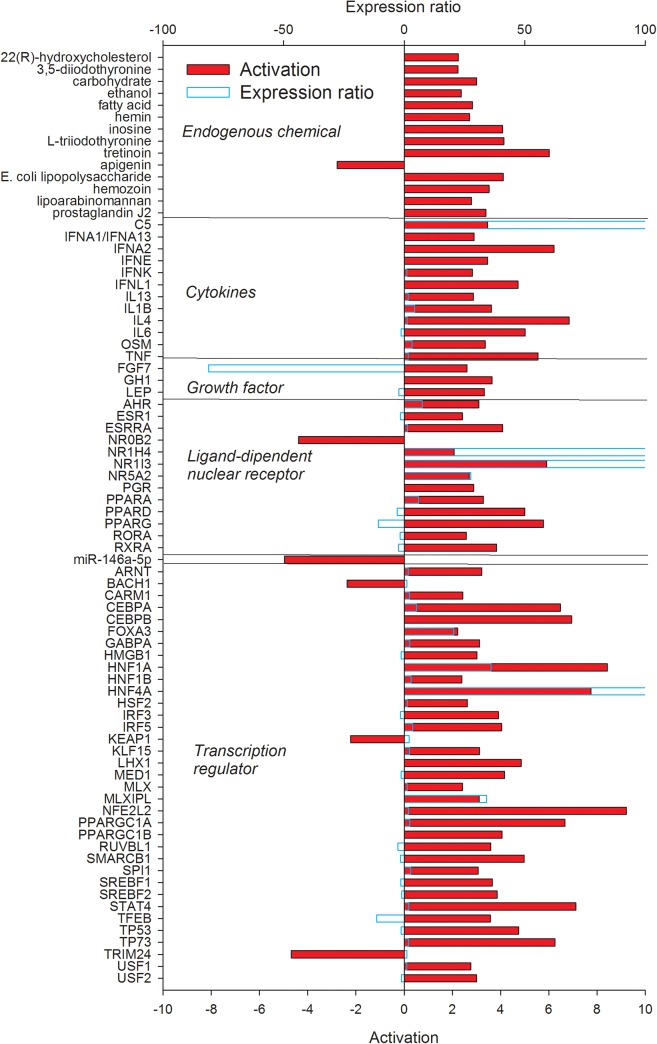
Upstream regulators (clustered in functional groups) of the DEG more expressed in liver *vs*. mammary tissue with an estimated Z-score ≥2. The Z-score is a prediction of the activation status of upstream transcriptional regulators using the molecular network that represent experimentally observed gene expression and are associated with a literature-derived regulation direction which can be either “activating” or “inhibiting” the DEG. Results were obtained using Ingenuity Pathway Analysis. Reported in light blue is the observed expression ratio of upstream regulators.

**Fig 6:**
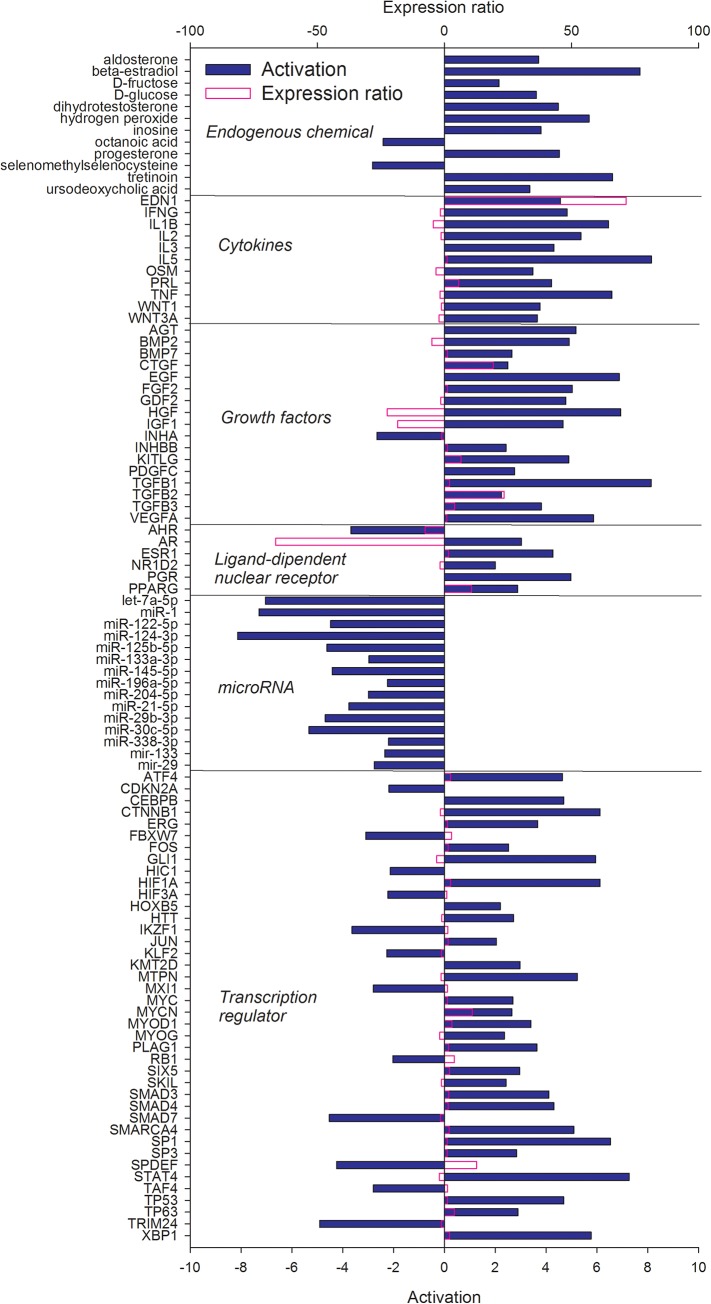
Upstream regulators (clustered in functional groups) of the DEG more expressed in mammary tissue *vs*. liver with an estimated Z-score ≥2. The Z-score is a prediction of the activation states of upstream transcriptional regulators using the molecular network that represent experimentally observed gene expression and are associated with a literature-derived regulation direction which can be either “activating” or “inhibiting” the DEG. Results were obtained using Ingenuity Pathway Analysis. Reported in purple is the observed expression ratio of up-stream regulators.

In an effort to identify potential upstream regulators that control expression of genes with different transcript abundance between liver and mammary tissue, we used IPA upstream regulator analysis. Upstream regulators are defined as any molecule that can affect the expression of genes, including transcription factors, growth factors, cytokines, microRNAs, and endogenous chemical. The activation state for each regulator is predicted by IPA based on global direction of changes of putative downstream regulated genes. The predicted activated or activating regulators including endogenous chemicals, cytokines, growth factors, transcription regulators, ligand-dependent nuclear receptors, and miRNA are shown in Figs 5 and 6 and a complete list (encompassing other categories of up-stream regulators) is provided in S4 File.

The upstream regulators of DEG more expressed in liver *vs*. mammary tissue included a large number of endogenous chemical, ligand-dependent nuclear receptors, and transcription factors (Fig 5). Almost all of these were estimated to be significantly activated (or activating the down-stream DEG) by IPA (i.e., with a Z score ≥2). Among endogenous chemicals IPA revealed several lipid molecules (e.g., the cholesterol-derivative hydroxycholesterol, tretinoin, fatty acids, and prostaglandin J2), thyroid hormones, glucose, and lipopolysaccharides (LPS). Among cytokines highly activated upstream regulators were estimated to be several interferons, the neutrophils chemoattractant C5, and several pro-inflammatory cytokines (e.g., IL1β, TNFα, and IL6). Few growth factors were revealed to activate downstream DEG more expressed in liver *vs*. mammary tissue, among these leptin and growth hormones. The importance of fatty acids in transcriptional regulation of liver metabolism is emerging in monogastrics [40]. The estimated importance of tretinoin, a commercial retinoic acid, indicate the importance of vitamin A for the transcriptional regulation of the liver, likely through PPAR [41]. The revelation of an important role of LPS in defining the transcriptomic difference between liver and mammary tissue is meaningful. The transcriptome of the liver in dairy cows is highly responsive to LPS [42] even only after 2.5 h after intramammary infusion of LPS [43]. Our data indicate that LPS has some role in shaping the transcriptome of the liver of healthy cows and prompt for inferring a microbiome-liver interaction.

Several ligand-dependent nuclear receptors (LdNR), which are known to control aspects of hepatic physiology and pathophysiology [44], were more induced in liver *vs*. mammary tissue and several had a higher expression in liver *vs*. mammary tissue. Among these were FXR (encoded by *NR1H4*) and constitutive androstane receptor (*NR1I3*), both responding to metabolites and xenobiotics [45], [46]. All peroxisome proliferator-activated receptor isotypes together with the RXR (although more expressed in mammary tissue, see S1 File) were estimated to be more activated in liver compared with mammary tissue, with PPARα being more expressed in liver *vs*. mammary tissue (Fig 5). Fatty acids are strong agonists of PPAR [37], [40], indicating that the liver is highly sensitive to fatty acid *via* PPAR but, potentially, also through LXR [46], which was 16-fold more expressed in liver *vs*. mammary tissue but was only numerically more activated in liver compared with mammary tissue (S4 File). All the LdNR were estimated to be highly induced in liver compared with mammary tissue with the exception of the Nuclear Receptor Subfamily 0 Group B Member 2 (*NR0B2*), an orphan receptor with activity in the inhibition of bile acid synthesis in liver [47].

The only up-stream miRNA of DEG more expressed in liver *vs*. mammary tissue which was estimated to be significantly inhibited by IPA was miR146a. Recently, it was demonstrated that this miRNA have anti-interferon activity in liver hampering the hepatocyte response to inflammation in human [48].

Thirty-five transcription regulators (TR) were estimated to actively participate in inducing the transcription of DEG more expressed in liver *vs*. mammary tissue (Fig 5); most of these were estimated to be activated. Several of the transcriptional regulators were related to lipid metabolism (especially cholesterol), such as *HNF1A*, *HNF4A*, *PPARGC1A*, *PPARGC1B*, *SREBF1*, and *SREBF2*. Other important transcription regulators were related to inflammation (e.g., *NFE2L2*, *CEBPB*), cell cycle (e.g., *CEBPA*, *TP53*), and glucose homeostasis (e.g., *MLXIPL*). The *HNF1A* [49] and *NFE2L2* [50] are known to participate in the proliferation of hepatocytes and *SMARCB1* is essential for hepatocyte differentiation [51]. *HNF4A*, an important transcription regulator in liver that can be also activated by acyl-CoA [46], was >300-fold more expressed in liver compared with mammary tissue and one of the most activated upstream transcription regulator in liver (Fig 5). The transcription factor *TRIM24*, important for liver homeostasis in mouse [52], *BACH1*, involved in the response to hepatic injury in rat [53], and *KEAP1*, important during liver regeneration [54], were overall more expressed in liver compared with mammary tissue but their activity was estimated to be overall inhibited in liver (Fig 5).

Among the upstream regulators of genes more expressed in mammary tissue *vs*. liver (Fig 6), there was a large number of endogenous compounds, including hormones, such as testosterone, and carbohydrates (e.g., glucose), estimated to significantly activate down-stream DEG, and octanoic acid, estimated to have an inhibitor effect. These data indicated that mammary tissue compared with liver is more under hormonal control, particularly progesterone, and the larger expression of some of the genes in mammary tissue *vs*. liver are also consequence of glucose, the precursor of lactose, one of the main milk compounds. The estimated inhibition of octanoic acid is not clear, considering that all the potential downstream target genes (e.g., *CD36*, *PPARG*, *FABP4*, see S4 File) were all more expressed in mammary tissue compared with liver (S1 File). Several cytokines were estimated to have a positive effect on the expression of mammary genes (Fig 6). Among these several are pro-inflammatory such as interferon gamma, IL1β, and TNFα; however, all with a lower expression in mammary tissue *vs*. liver (Fig 6 and S1 File). Other cytokines were expected to have a positive effect on mammary tissue *vs*. liver, such as prolactin and endothelin 1, which were also more expressed in mammary tissue *vs*. liver (Fig 6).

Different than the liver (Fig 5), IPA estimated a large importance of growth factors in controlling the transcription of genes more expressed in mammary tissue *vs*. liver (Fig 6). With the exception of the Inhibin Alpha (*INHA*), a protein involved in the regulation of organogenesis in mammary gland [55], all the growth factors were deemed to have an activating effect on down-stream DEG. Among these were several transforming growth factor isoforms, known to be important for mammary epithelial differentiation, at least as demonstrated for TGFβ [56]. Insulin-like growth factor 1 and hepatic growth factors, both more expressed in liver compared with mammary tissue, have a positive influence on the expression of DEG that were more transcribed in mammary tissue compared to liver. Insulin-like growth factor 1 (IGF1), more expressed in liver, was one of the significantly activated upstream regulators in mammary tissue (Fig 6). The IGF1 plays a pivotal role in mammary development [57] and in controlling proliferation and milk protein synthesis of lactating bovine mammary tissue [58], [59], [60]. The larger production of IGF1 by the liver might indicate this hormone being a principal signaling molecule involved in the liver-mammary crosstalk.

Compared with liver the mammary transcriptome was estimated to be under control of fewer LdNR (Fig 6). With the exception of aryl hydrocarbon receptor (*AHR*), which is important for the transcriptional control of xenobiotic-related genes and its activation is associated with inhibition of lactation in human mammary epithelial cells [61], all the LdNR were estimated to be activated in mammary tissue. Among these PPARγ, progesterone receptor, and estrogen receptors were strongly activated. The importance of PPARγ in mammary tissue is likely related to the role in controlling transcription of genes coding for proteins involved in milk fat synthesis [37], while a role of progesterone and estrogen in mammary development and lactation is well established [58].

Different than the genes more expressed in the liver compared to mammary tissue, where only one miRNA was estimated to be important (Fig 5), the genes more expressed in mammary tissue compared to liver are targets of a larger number of miRNAs. Among the likely more inhibited, the miRNA29b has strong epigenetic effects on the expression of several lactogenic genes [62], let-7a-5p is highly expressed in cow mammary [63], and miRNA1 has a strong control in the expression of β-lactoglobulin [64].

A relatively large number of transcription factors were estimated to have had an impact on the expression of genes more abundant in mammary tissue *vs*. liver. Several transcription factors estimated to be activated are related to cell cycle (e.g., *TP53*), cell adhesion (e.g., *CTNNB1*), protein synthesis (e.g., *MYC*), and endoplasmic reticulum stress (e.g., *ATF4*, *XBP1*) while the ones estimated to be inhibited are mostly related to negative regulation of proliferation (e.g., *TRIM24*, *FBXW7*). The *SPDEF*, involved in regulation of progenitor cells and breast cancer development [65], was more expressed in mammary tissue compared with liver but was estimated to be overall inhibited (Fig 6).

### Potential crosstalk between liver and mammary tissue

The liver and the mammary gland have complementary metabolic roles during lactation in cow [66]. Glucose synthesized by the liver is released into the circulation and is taken up by the mammary gland mostly to produce lactose. Few additional relationships are known to exist between liver and mammary tissue, with a dominant role of the liver over mammary. One of such example is the IGF1. This hormone is primarily produced by the liver and it is known to play important roles in mammary gland development and differentiation [59], [67] and milk protein synthesis [60], [68]. Previous analysis of the transcriptome adaptation of the mammary, liver, and adipose tissue during the peripartum in dairy cows uncovered a reciprocal metabolic adaptation between adipose and mammary tissue, but the data did not uncover a coordinated metabolic adaptation between mammary tissue and liver, with the exception of the contemporaneous increase in gluconeogenesis of the liver and milk lactose synthesis in mammary tissue [8].

**Fig 7:**
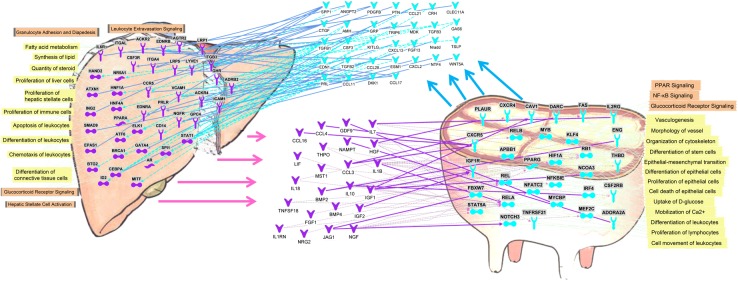
Potential crosstalk between liver (purple objects, arrows, and lines) and mammary tissue (blue objects, arrows, and lines) obtained by *in silico* approach via Ingenuity Pathway Analysis. Genes coding for secreted proteins (i.e., cytokines, growth factors) with higher expression (≥2-fold; FDR<0.001) in liver *vs*. mammary tissue have the potential to interact with receptors more expressed (≥2-fold; FDR<0.001) in mammary tissue *vs*. liver and *vice versa*. The most significant functions (yellow shade) and pathways (orange shade) associated with the receptors are reported.

The simultaneous presence of transcriptomic data from the liver and mammary tissue of the same animals can allow uncovering the cross talk between the two tissues *via in silico* approaches a previously carried out in mammary parenchyma and fat pad in growing dairy calves [12]. The potential crosstalk between liver and mammary tissue is depicted in Fig 7. The network analysis using IPA uncovered 32 signaling molecules (i.e., cytokines and growth factors) among DEG expressed >2-fold in mammary tissue compared with liver and, thus, with a potential higher production and release by the mammary tissue. These signaling molecules are potentially able to interact directly or indirectly with 42 among receptors and transcription factors more expressed in liver compared with mammary tissue. These include also 3 LdNR: PPARα, androgen receptor (*AR*), and Nuclear receptor 5A (*NR5A*). The latter plays a pivotal role in controlling steroidogenic enzymes [69]. The functional analysis of the receptors and transcription factors potentially affected by the signaling molecules coming from the mammary tissue uncovered a significant enrichment of functions related to the control of lipid metabolism, immune function, and hepatic proliferation. Among the signaling molecules potentially released by the mammary, the osteopontin (*SPP1*), one of the most expressed genes in mammary tissue during lactation [30], and the connective tissue growth factor (*CTGF*), with a known fibrogenic activity in liver [70] while in mammary of mouse has lactogenic properties [71], had the highest number of interactions with the receptors more expressed in liver compared with mammary tissue (Fig 7).

The IPA analysis uncovered 23 cytokines and growth factors more expressed in the liver with the capacity to interact with 31 receptors and transcription factors, including the PPARγ, more expressed in mammary tissue *vs*. liver (Fig 7). Among the signaling molecules the analysis uncovered the presence of *IGF1* and *IGF2*, which should be expected, but also several other growth factors (e.g., *GDF9* or growth differentiation factor 9 and *HGF* or hepatocyte growth factor) and cytokines/chemokines, such as *CCL4*, *IL1B*, and *BMP2*. The functional analysis of the receptors sensing the above signaling molecules in mammary tissue uncovered a significant enrichment of functions related to vasculogenesis, innate immune cells and inflammation (i.e., NFκB pathway), and differentiation and proliferation of epithelial cells. From metabolic standpoint the liver appeared to partly control mammary uptake of glucose and calcium and lipid metabolism (Fig 7).

Overall, the analysis uncovered a potential large crosstalk between liver and mammary tissue with a reciprocal control of metabolism, differentiation, proliferation, and regulation of immune function. The analysis allowed proposing that the mammary has a control (likely induction) of lipid metabolism of the liver while the liver has some control over mammary utilization/synthesis of three important constituents of the milk: lactose, calcium, and fat.

The large crosstalk regarding the innate immune system uncovered by the analysis is indicative of a reciprocal role of the two organs in coordinating a potential response to infection, such as mastitis. An important role of the liver and the liver and mammary crosstalk during mastitis was uncovered by transcriptomic works in dairy cows [42] [13].

## Conclusions

In this study, the biological differences between liver and mammary tissue during lactation were detected at the level of transcription in mid-lactation dairy cows. The transcriptome analysis revealed that in dairy cows the liver has a larger metabolic capacity compared with mammary tissue. This difference appears to be regulated also by a plethora of upstream regulators, including endogenous compounds and transcription factors. Mammary tissue *vs*. liver has an apparent larger protein synthesis capacity, a larger development and proliferation, and higher communication ability. The latter was also confirmed by the large importance of growth factors determining the observed DEG more expressed in mammary tissue *vs*. liver. Overall the analysis identified an extremely large functional difference between liver and mammary tissue and exposed potential factors decisive for such difference.

The *in silico* analysis of the crosstalk between liver and mammary tissue uncovered a relatively large communication between the two organs with a reciprocal control of lipid metabolism and development/proliferation. The analysis allowed identifying previously unknown role in the crosstalk between liver and mammary tissue of several signaling molecules. For instance the data indicate that SPP1 and CTGF may be among the most important signaling molecules used by the mammary tissue to communicate with the liver; however, the effect of these signaling molecule in liver remains to be clarified. Similarly, the data indicated that the liver potentially can affect the mammary biology by a relatively large number of signaling molecules affecting the metabolism and proliferation of the mammary tissue. Therefore, the present analysis provides a large number of novel potential candidates for future targeted crosstalk studies. The crosstalk analysis also uncovered a reciprocal control of the innate immune activity of the two organs. This can have important implications for the response to mastitis.

## Supporting information

S1 File Complete dataset with statistical results and annotations. (XLSX)

S2 File Complete results from the Dynamic Impact Approach analysis of the KEGG pathways plus analysis of chromosomes. (XLSX)

S3 File Results from DAVID analysis of all DEG and DEG with ≥2-fold expression ratio cut-off between liver and mammary tissue. (XLSX)

S4 File Ingenuity Pathway Analysis results of enriched pathways, functions, and upstream regulators. (XLSX)

S5 File Results of geNorm analysis to identify reliable internal control genes (XLSX)

S6 File Primer-pairs sequences for Real-Time PCR analysis. (XLSX)

S1 Fig Outlier Box Plots to identify biological outliers. All the animals had a similar distribution of the data and none was considered outlier. (TIF)

S2 Fig Scatter plot of differentially expressed genes (DEG) between liver and mammary tissue with a FDR<0.001. Indicated are some genes of interest among the ones most expressed in one tissue *vs*. the other. (PNG)

S3 Fig Sixty differentially expressed genes (DEG; FDR≤0.001) with the higher difference in expression between liver and mammary tissue. (TIF)

S4 Fig Comparison between RTqPCR and microarray in expression of 9 selected transcripts. All selected transcripts were significant different between the two tissues in microarray analysis. All tested genes were differentially expressed also using RTqPCR
(TIF)

S5 Fig ‘Galactose metabolism’ KEGG pathway visualization using KegArray tool (available at http://www.kegg.jp/kegg/download/kegtools.html). Orange-red shaded objects denote genes more expressed in liver *vs*. mammary tissue; green shaded objects denote genes more expressed in mammary tissue *vs*. liver.
(PNG)

S6 Fig Visualization of ‘Insulin signaling pathway’. See legend for S5 Fig for details.
(PNG)

S7 Fig Visualization of ‘PPAR signaling pathway’. See legend for S5 Fig for details.
(PNG)

S8 Fig Visualization of ‘Adipocytokines signaling pathway’. See legend for S5 Fig for details.
(PNG)

S9 Fig DAVID results of Gene Ontology (GO) Biological process terms enriched in differentially expressed genes (FDR≤0.001) with >2-fold expression between liver and mammary tissue. Upper panel genes more expressed in liver *vs*. mammary tissue and lower panel genes more expressed in mammary tissue *vs*. liver. The GO results (see S3 File) were reduced and visualized using REVIGO tool (available at http://revigo.irb.hr/).
(PNG)

S10 Fig DAVID results of Gene Ontology (GO) Cellular component terms enriched in differentially expressed genes (FDR≤0.001) with >2-fold expression between liver and mammary tissue. Upper panel genes more expressed in liver *vs*. mammary tissue and lower panel genes more expressed in mammary tissue *vs*. liver. The GO results (see S3 File) were reduced and visualized using REVIGO tool (available at http://revigo.irb.hr/).)
(PNG)

S1 Table Ingredients and composition of experimental diets. (DOCX)
