# Supplementary figures and images for: Transcriptome difference and potential crosstalk between liver and mammary tissue in mid-lactation primiparous dairy cows
Source: PLoS One. 2017 Mar 14;12(3):e0173082. doi: 10.1371/journal.pone.0173082 (PMC5349457; doi:10.1371/journal.pone.0173082)

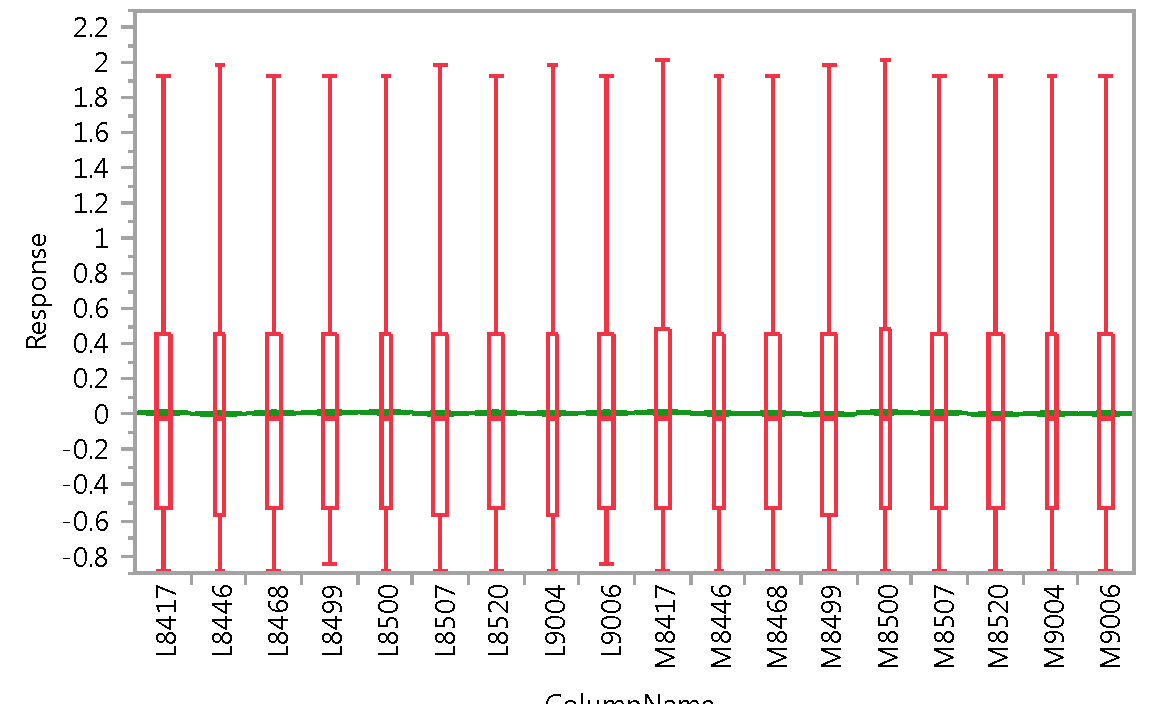

Supplement: S1 Fig — (TIF) [file pone.0173082.s007.tif]

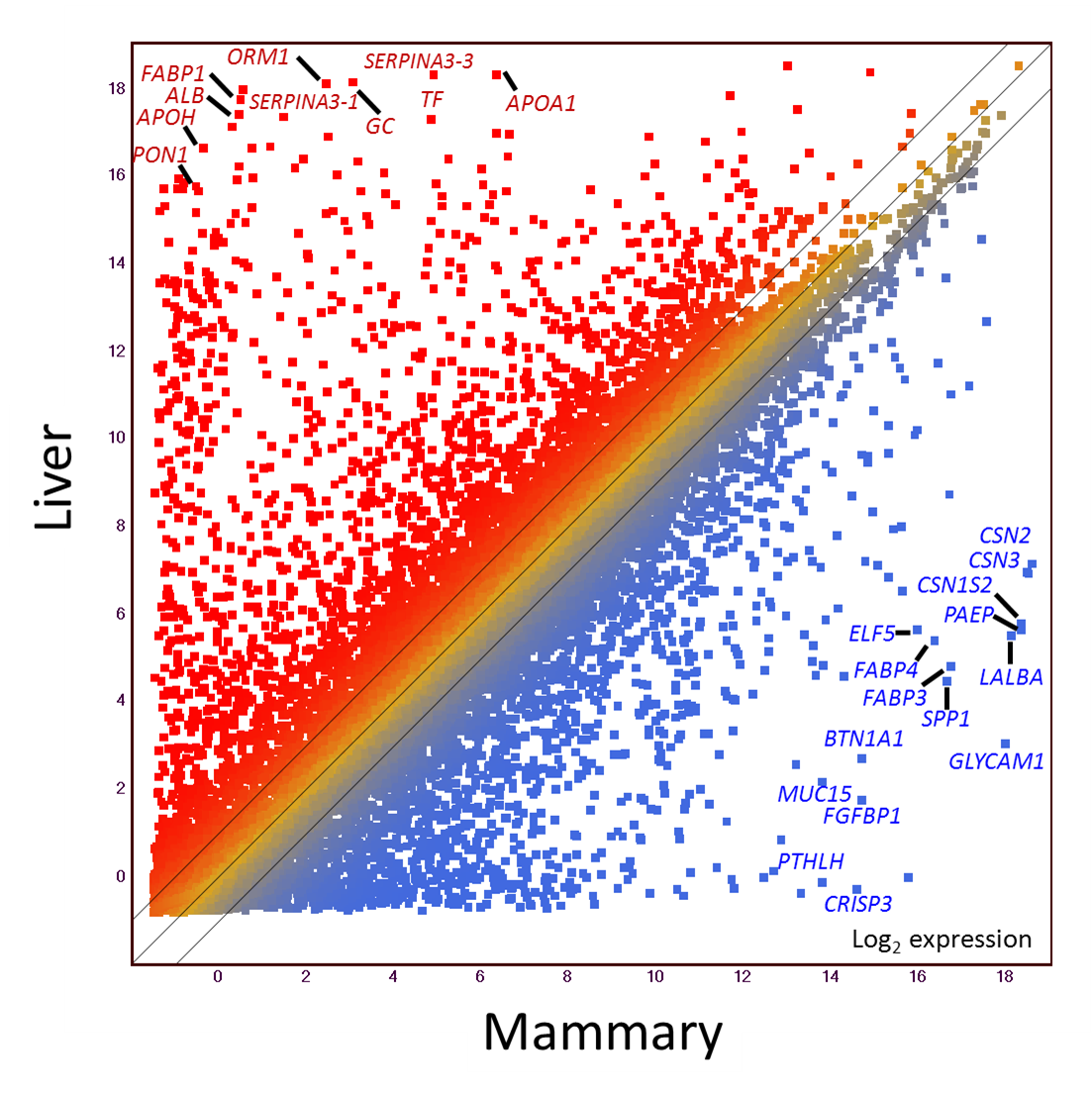

Supplement: S2 Fig — (PNG) [file pone.0173082.s008.png]

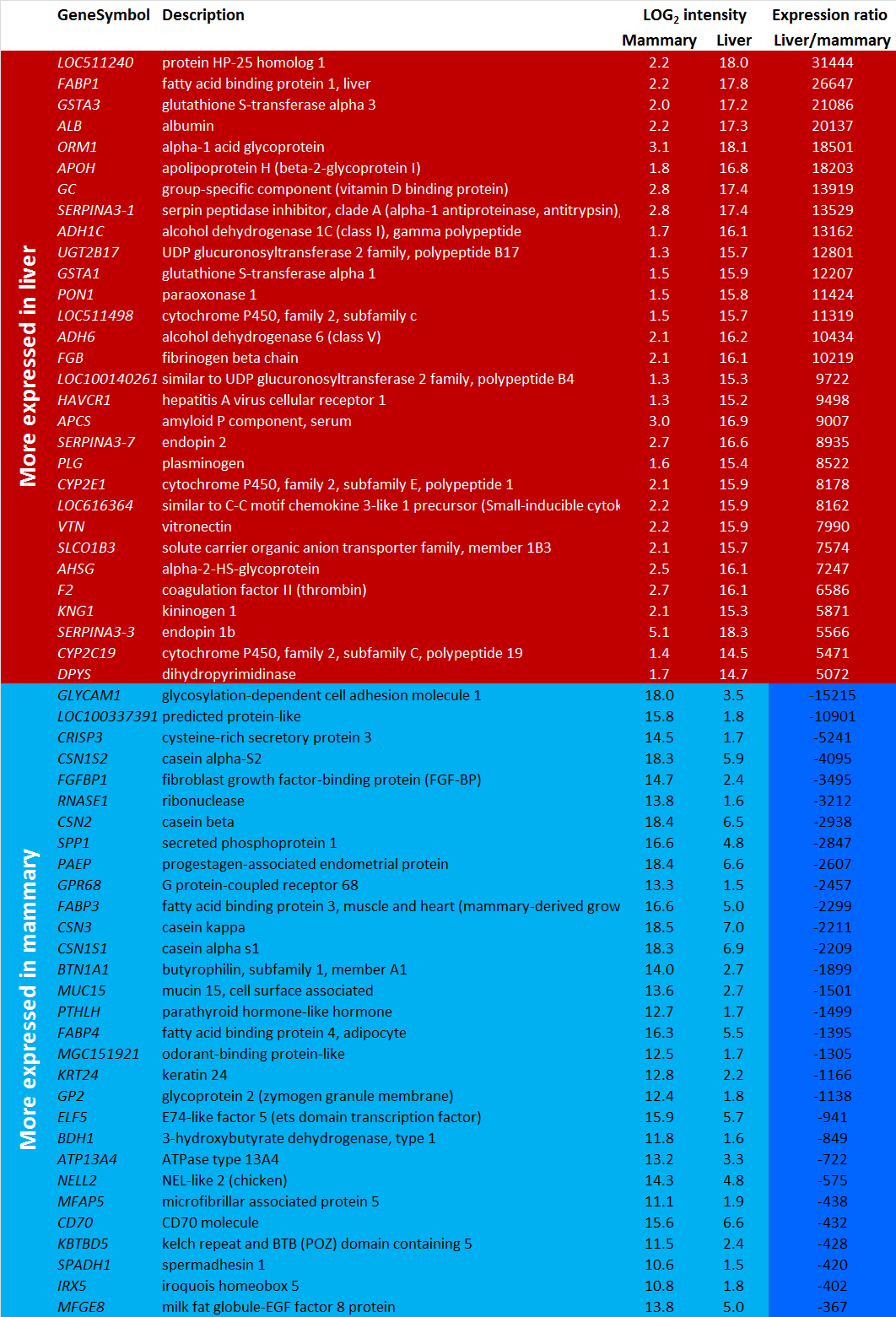

Supplement: S3 Fig — (TIF) [file pone.0173082.s009.tif]

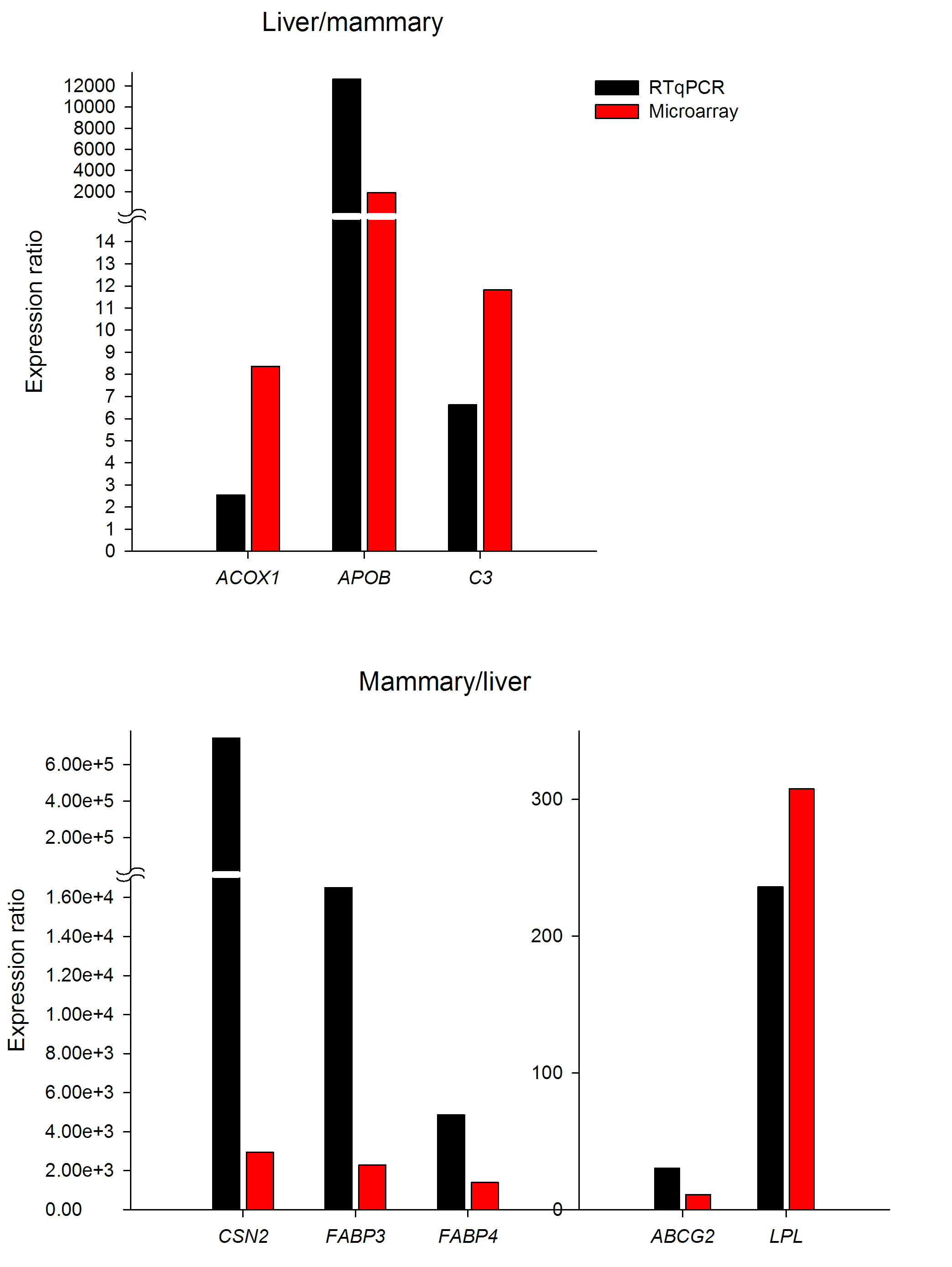

Supplement: S4 Fig — All selected transcripts were significant different between the two tissues in microarray analysis. All tested genes were differentially expressed also using RTqPCR (TIF) [file pone.0173082.s010.tif]

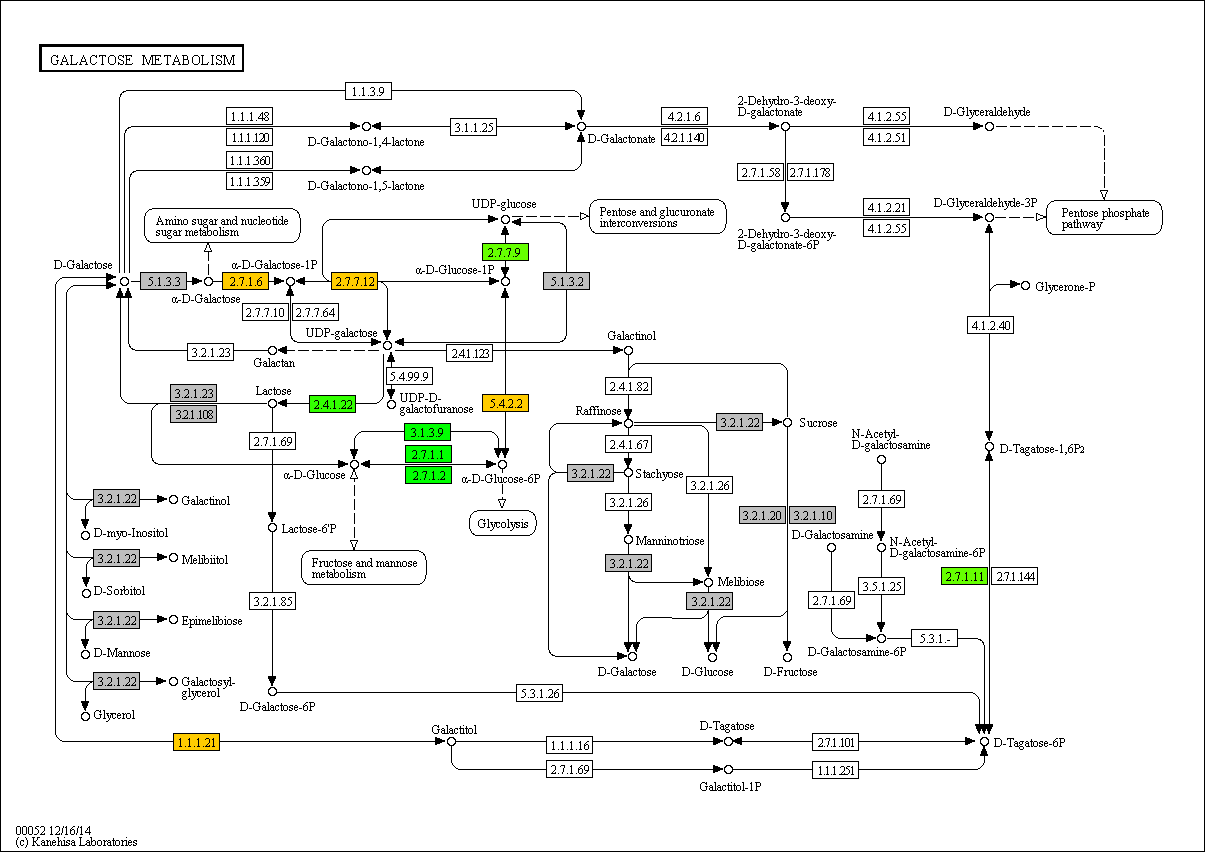

Supplement: S5 Fig — Orange-red shaded objects denote genes more expressed in liver vs. mammary tissue; green shaded objects denote genes more expressed in mammary tissue vs. liver. (PNG) [file pone.0173082.s011.png]

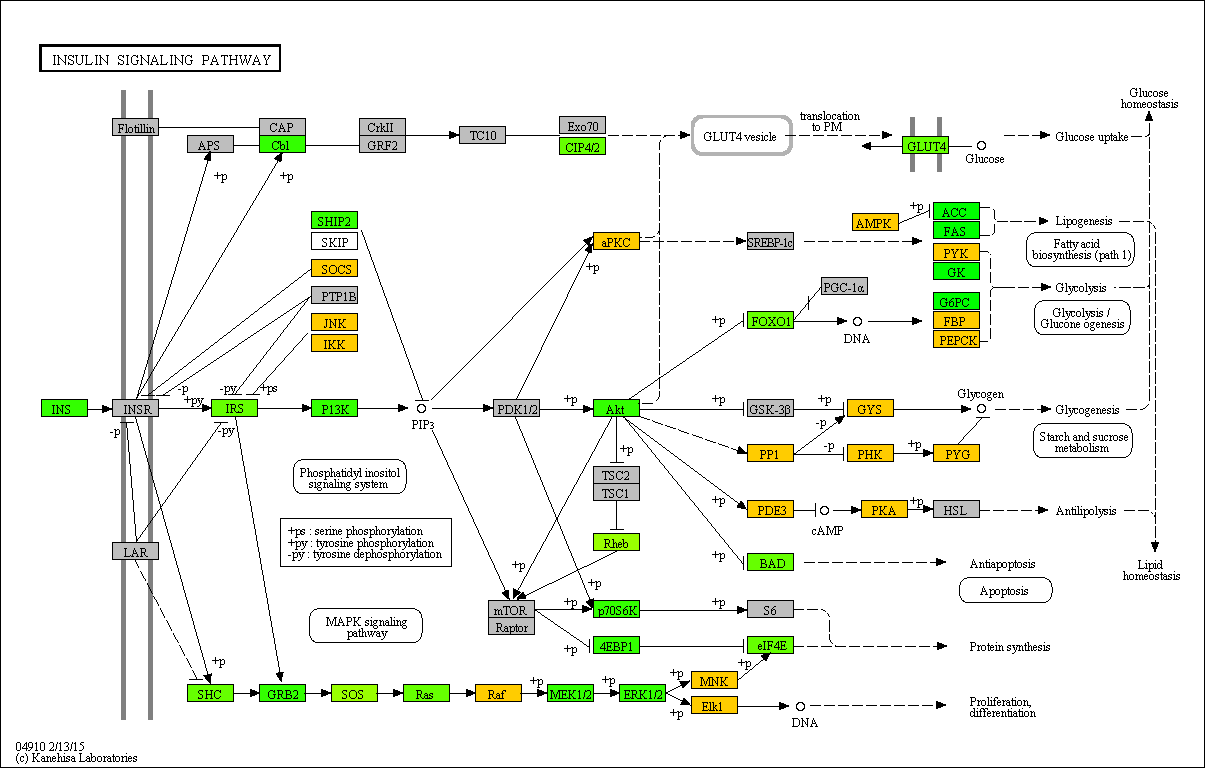

Supplement: S6 Fig — See legend for S5 Fig for details. (PNG) [file pone.0173082.s012.png]

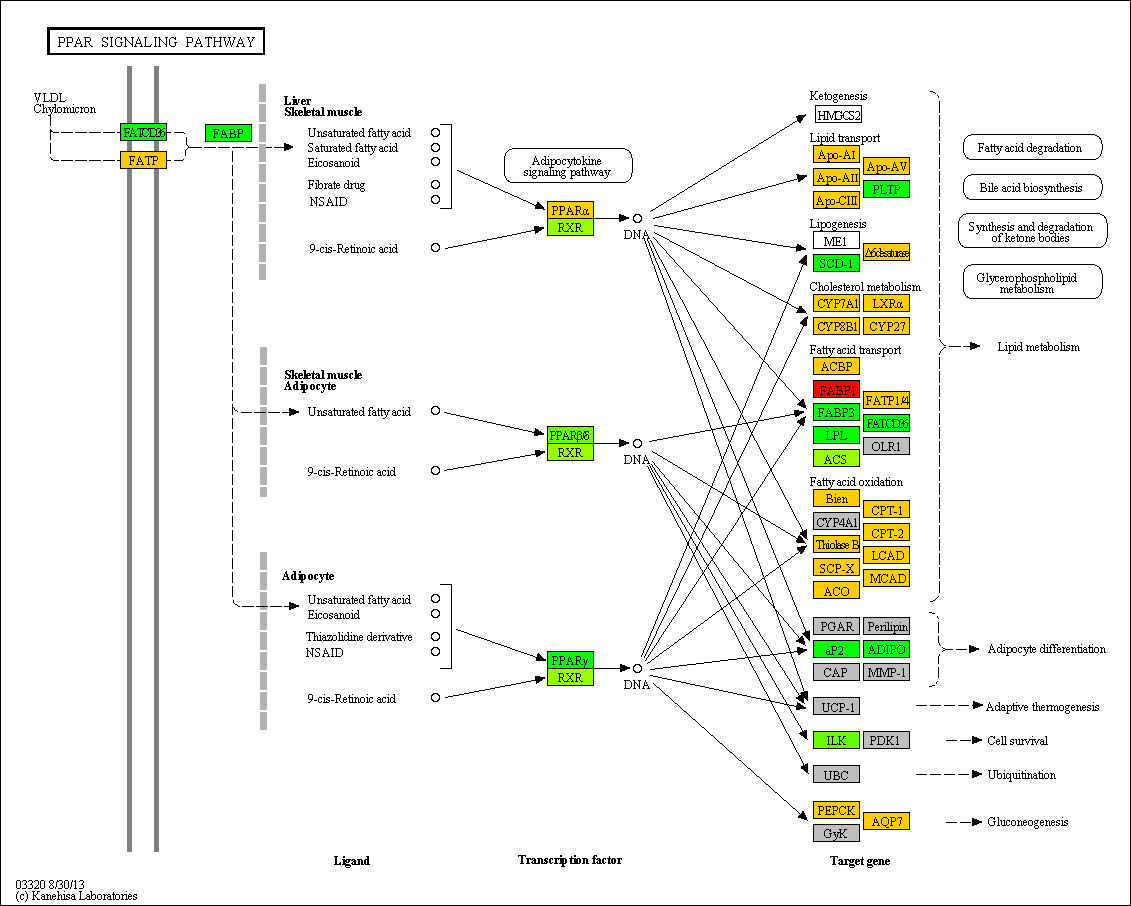

Supplement: S7 Fig — See legend for S5 Fig for details. (PNG) [file pone.0173082.s013.png]

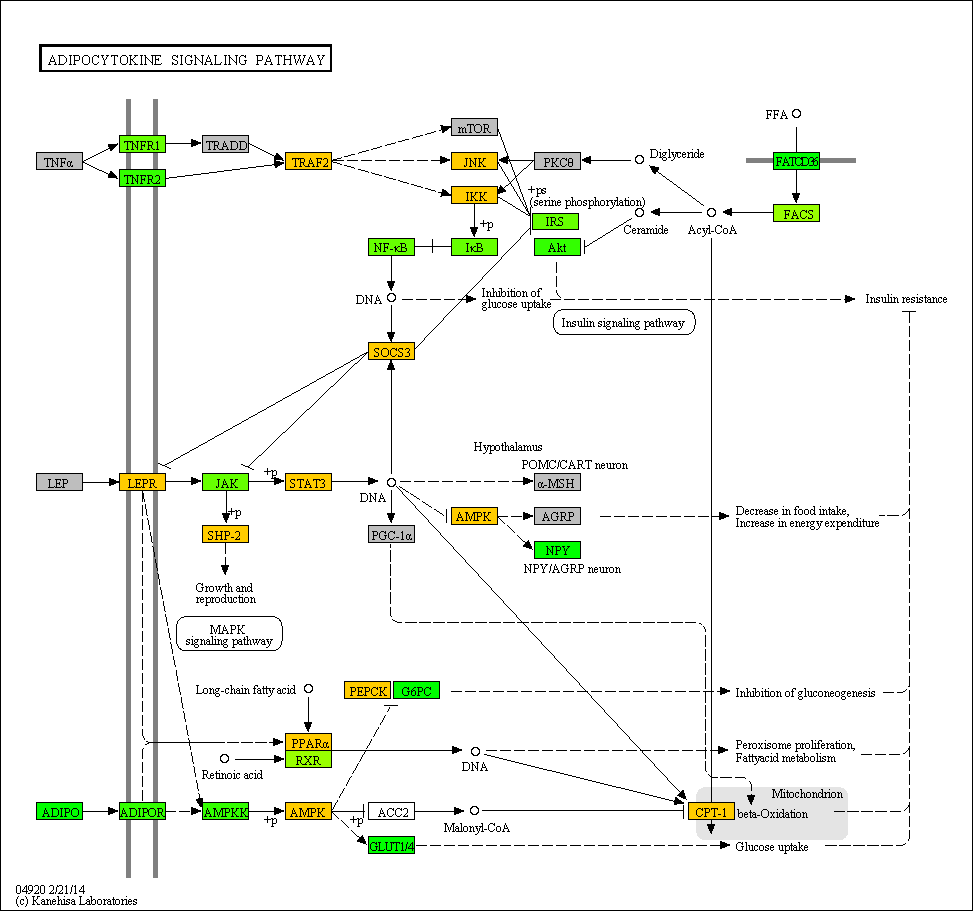

Supplement: S8 Fig — See legend for S5 Fig for details. (PNG) [file pone.0173082.s014.png]

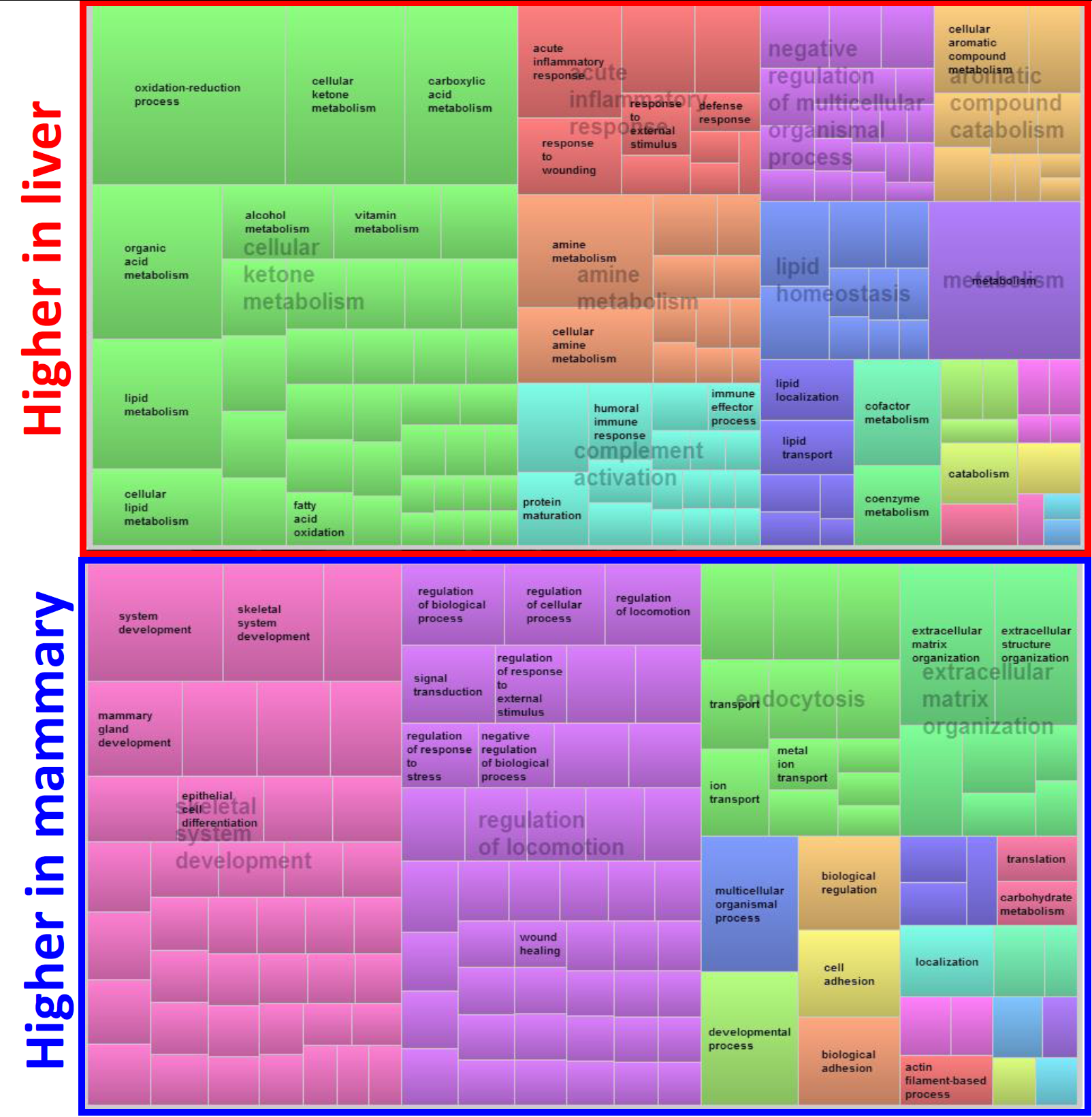

Supplement: S9 Fig — Upper panel genes more expressed in liver vs. mammary tissue and lower panel genes more expressed in mammary tissue vs. liver. The GO results (see S3 File) were reduced and visualized using REVIGO tool (available at http://revigo.irb.hr/). (PNG) [file pone.0173082.s015.png]

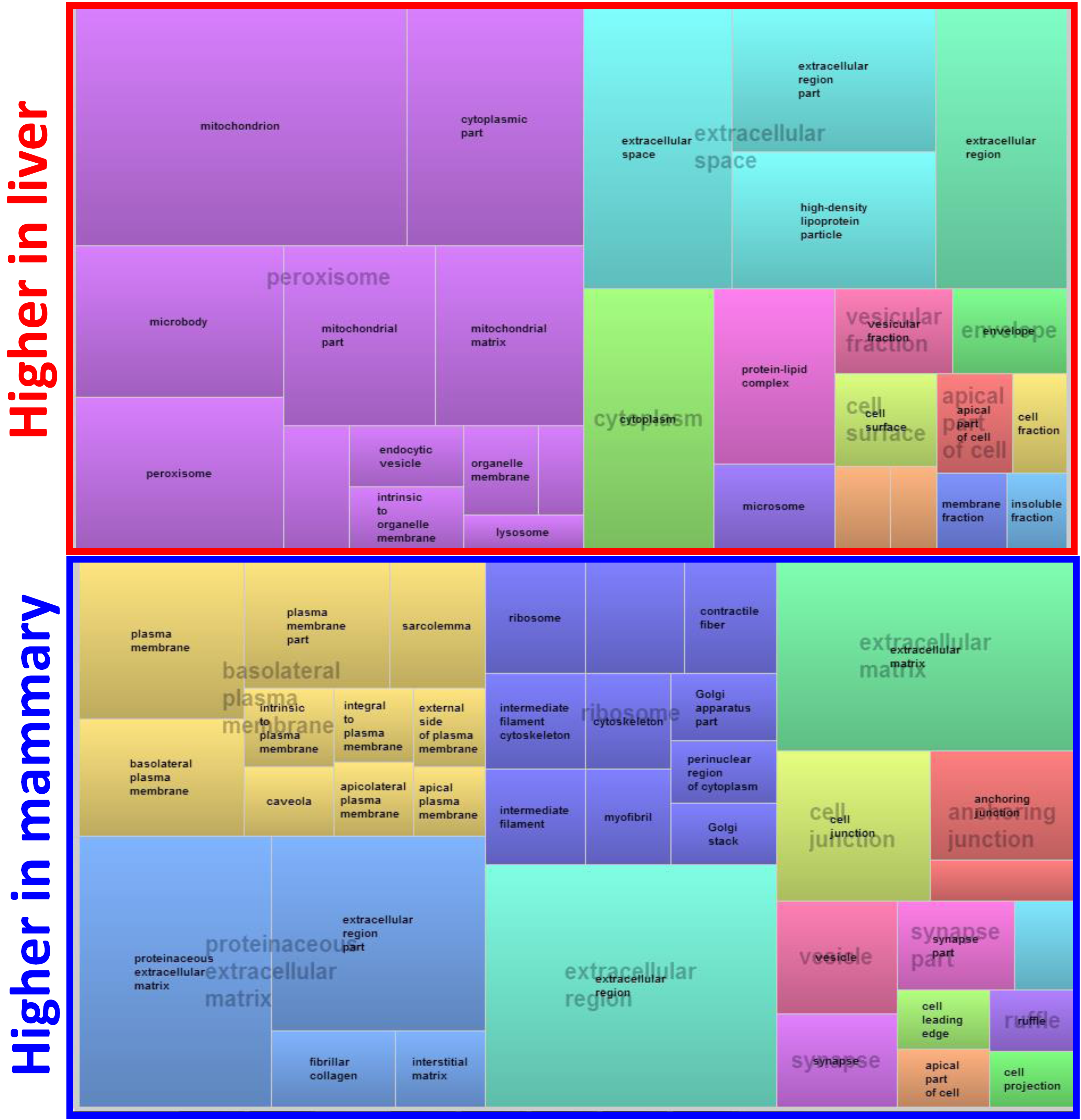

Supplement: S10 Fig — Upper panel genes more expressed in liver vs. mammary tissue and lower panel genes more expressed in mammary tissue vs. liver. The GO results (see S3 File) were reduced and visualized using REVIGO tool (available at http://revigo.irb.hr/).) (PNG) [file pone.0173082.s016.png]
