# Supplementary material for: Transcriptome difference and potential crosstalk between liver and mammary tissue in mid-lactation primiparous dairy cows
Source: PLoS One. 2017 Mar 14;12(3):e0173082. doi: 10.1371/journal.pone.0173082 (PMC5349457; doi:10.1371/journal.pone.0173082)
Supplement: S1 Table — (DOCX) [file pone.0173082.s017.docx]

Table S1. Ingredients and composition of experimental diets

| Ingredients [g/100 g of DM] | CS (F:C = 40:60)^1^ | MF (F:C = 60:40)* |
| --- | --- | --- |
| Corn stover | 37.1 | - |
| Alafalfa hay | - | 28.4 |
| Corn silage | - | 26.5 |
| Chinese wild rye | - | 3.7 |
| Corn | 33.5 | 22.8 |
| Wheat bran | 3.0 | - |
| Soybean meal | 23.6 | 11.8 |
| Cottonseed fuzzy | - | 5.1 |
| Calcium phosphate | 0.4 | 0.6 |
| Limestone | 1.3 | - |
| NaCl | 0.5 | 0.5 |
| Mineral-vitamin mix^2^ | 0.6 | 0.6 |
| Chemical composition [g/100 g of DM] |  |  |
| Dry matter content | 54.47 | 55.78 |
| Crude protein | 16.90 | 16.70 |
| Neutral detergent fibre | 41.01 | 44.18 |
| Acid detergent fibre | 21.15 | 26.06 |
| Ether extract | 1.58 | 2.24 |
| Calcium | 0.89 | 0.82 |
| Phosphorus | 0.21 | 0.31 |
| Net energy lactation (NE_L_)^3^ [MJ/kg of DM] | 6.19 | 6.35 |

Notes: ^1^F:C, Forage to concentrate ratio;

^2^Containing (per kilogram dry matter of premix): vitamin A 2,000,000 IU, vitamin D 600,000 IU, vitamin E 10,800 mg, ferrum 4,080 mg, copper 4,989 mg, zinc 180 mg, manganese 17,500 mg; cobalt 8,805 mg;

^3^Estimated based on chemical compositions and 24 h gas production of diets (Hohenheim gas test).
